# What Cochrane means now

**DOI:** 10.1002/14651858.ED000179

**Published:** 2026-08-27

**Authors:** Rupa Sarkar

**Affiliations:** Cochrane


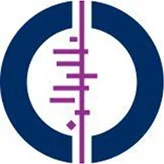


Never has there been so much information available, and never has it been harder to know what to trust. We face an expanding tide of ‘AI slop’, computer‐generated content, produced at scale, largely unverified, sometimes persuasive, and frequently wrong. The need for trusted, rigorous, independent evidence is becoming existential. It is in this context that I am stepping into the role of Editor in Chief of Cochrane.

As health care faces new challenges and unprecedented opportunities, Cochrane is beginning a new editorial series examining some of the defining issues of our time, from misinformation and artificial intelligence (AI), to bias, competing interests and trust in research. These pieces are an invitation to think together about what Cochrane is, what it must become, and how we ensure that our high‐quality evidence continues to improve lives by supporting better decisions in health.

My own view of change was shaped early, and not in a way I found particularly edifying at the time. I spent years developing mouse models for a developmental disorder — painstaking, expensive and slow work — only to find out at the end that newer technologies could now achieve similar results in weeks. I was furious. But what I took from my time as a scientist was that progress rarely moves in a straight line, and that being certain you're doing the right thing while being sceptical that it will work are not diametrically opposed positions. Both can be true simultaneously. I carry both views into this role: genuine optimism that AI can help build a more truthful, rigorous, and equitable future, and genuine scepticism about the gap between what is claimed and what AI currently delivers.

Cochrane has often sat close to such tensions. For decades, the Cochrane community has driven some of the most significant improvements in human health by championing systematic reviews and advancing the methods behind evidence synthesis. But if we are to remain relevant, we need to be precise about what Cochrane means now.

Cochrane is more than a producer of systematic reviews. It is an evidence activist organization that actively defends the quality, integrity, and essential use of evidence in public discourse.

We've seen what happens when that defence is absent. Interventions for COVID‐19 [Bibr ED000179-bib-0001 ED000179-bib-0002], and Alzheimer's disease [Bibr ED000179-bib-0003], or approaches to antibiotic prescribing [Bibr ED000179-bib-0004], gained widespread acceptance before careful evidence synthesis exposed how little benefit they provided, and in some cases the harm they caused. Those conclusions were not popular at the time they were needed most. That is precisely Cochrane's job; to be the institution willing to give the unwelcome message before the wave of confidence breaks, not after. Living up to that ideal means we have to both reaffirm and evolve our mission. There are five ways in which I want to do that, though I admit that the framing that follows is neater than the reality, which will always be messier and more iterative.

First, we strengthen editorial leadership and accountability. Trust depends not only on rigorous methods but also on clear responsibility for decisions. That means consistent, transparent, strategically aligned editorial judgement, applied earlier in the process and concentrated where expertise genuinely adds value, not spread thinly across every stage in a way that slows everything down without improving anything. We've started making improvements, but this remains a work in progress.

Second, we move faster without cutting corners that could affect our trustworthiness. I am frequently asked about this tension, and I don't actually think it is one. Slow evidence fails just as badly as sloppy evidence. Our editorial roadmap is built around increasing our speed, with clearer accountability, earlier decision points, fewer unnecessary revision rounds, and AI tools that will support the process, not replace the judgement at the heart of it. We're investing in ensuring our evidence is up to date, so our conclusions stay current as new data come in. Median time from submission to publication in 2026 has come down from 11.9 months in January to 8.6 months in July. Our next target is 6 months. That's ambitious but achievable if we stop duplicating effort where it doesn't protect quality.

Third, we lead in methodology, not only for synthesis but across the full lifecycle of evidence generation. Cochrane's expertise in appraising primary research puts us in a position very few organizations share. We can help shape the standards by which research is produced in the first place, not just evaluated afterwards. That means being at the table when trials are designed, when AI tools are validated, when reporting guidelines are written. Our study on AI in evidence synthesis is part of this development [Bibr ED000179-bib-0005], as is our new approach to managing retracted publications [Bibr ED000179-bib-0006]. We'll explore opportunities to expand the scope of what we publish, including innovative approaches to producing research and evidence, so they benefit from the same rigorous, transparent scrutiny and community engagement that underpin all Cochrane content.

Fourth, we need to be clearer about what counts as credible evidence and more strategic about what we commission. Randomised controlled trials remain foundational, but no single study design can answer every question. To meet the needs of patients, clinicians, and policymakers, we need to draw on the forms of evidence that are most appropriate for the decisions at hand. Target trial emulation, observational studies and prognostic modelling all have a role when applied rigorously, and Cochrane is leading that methodological conversation. At the same time, our current pipeline is dominated by intervention review questions. This tells me we need to be more deliberate about identifying the questions that most need answering in global health, considering the evidence that is needed to answer them, and actively commissioning reviews to address them.

Fifth, and most importantly, Cochrane is a community first. Our greatest strength lies in the global network of thousands of researchers, clinicians, methodologists, and public contributors who build this work. Right now, some of them feel they're on the outside looking in. I hear this in conversations about centralization, about decisions that feel opaque, about distance from the centre. The Central Editorial Team exists to support the community in producing evidence that has the impact it deserves. In practice, that means co‐designed processes, transparency about how decisions are made, and building future leadership, particularly from the Global South, to address historical disparities in evidence production. This series is an invitation to collaborate on shaping this future together.

Cochrane has helped drive some of the greatest advances in global health, and matters now more than at any point in its history; not as a relic of a slower information age, but as one of the few institutions built for this one. I believe we can lead the next leap in the production of trusted, rigorous and independent evidence by evolving thoughtfully, critically, and together.

Ultimately, this work is never only about evidence, it is about the kind of world we want to build, and the one we leave behind.

## End Notes

**Declarations of interest**

RS is employed by Cochrane as Editor in Chief.

**Acknowledgments**

The author would like to acknowledge and thank Karla Soares‐Weiser (CEO, Cochrane), Toby Lasserson (Deputy Editor in Chief, Cochrane), Harry Dayantis (Head of Communications, Cochrane) and Anne Flegel (Deputy for Editorial Operations, Cochrane) for their support during the development of this editorial. The author would also like to acknowledge and thank Denise Mitchell (Senior Production Editor, Cochrane) and Elizabeth Royle (Production Manager, Cochrane) for copy editing, and Justin Mann (Managing Editor, Cochrane) for managing the editorial process.

**Provenance and peer review**

This editorial was originated by the Editor in Chief and was not externally peer reviewed.
